# A new strategy for prenatal genetic screening of copy number variations in the *DMD* gene: A large cohort study based on NIPT analysis

**DOI:** 10.1002/ctm2.1706

**Published:** 2024-05-26

**Authors:** Yan Wang, Yan Sun, Lulu Meng, Quanze He, Jingyu Zhao, Ran Zhou, Zhonghua Wang, Jianxin Tan, Dingyuan Ma, Linlin Fan, Yunmei Chen, Yuguo Wang, Zhu Jiang, Zhihong Qiao, Xiaojuan Wu, Binbin Shao, Ying Xue, Lijie Song, Ting Wang, Ping Hu, Zhengfeng Xu

**Affiliations:** ^1^ Department of Prenatal Diagnosis Women's Hospital of Nanjing Medical University Nanjing Women and Children's Healthcare Hospital Nanjing China; ^2^ BGI Genomics Shenzhen China; ^3^ Center for Reproduction and Genetics The Affiliated Suzhou Hospital of Nanjing Medical University Suzhou Municipal Hospital, Gusu School, Nanjing Medical University Suzhou China; ^4^ Clin Lab BGI Genomics Nanjing China; ^5^ Clin Lab BGI Genomics Tianjin China

Prenatal testing (NIPT) has been widely used in clinical screening for foetal chromosomal imbalances.^1^ Although the same NIPT data may be used to identify maternal copy number variants (CNVs),^2^ very limited information on CNVs in the *DMD* gene is available for large‐scale populations. Here, we developed a new strategy for prenatal screening of CNVs in the *DMD* gene using NIPT data. Using this strategy, we evaluated the feasibility of using NIPT data to detect maternal CNVs in the *DMD* gene in a large cohort of 135,047 pregnant women. In addition, the carrier rate as well as the spectrum and types of maternal CNVs in the *DMD* gene were assessed.

In this study, we implemented a self‐developed method for detecting maternal CNVs. We reanalysed 135,047 NIPT samples collected from Nanjing Maternity and Child Health Care Hospital and Suzhou Municipal Hospital between January 2017 and December 2021 to identify maternal CNVs in the *DMD* gene (Figure S1). A total of 224 maternal CNVs in the *DMD* gene were identified (Table S1). Among these 201 CNVs (177 exonic and 24 intronic) validated by multiplex ligation‐dependent probe amplification (MLPA), 128 true‐positive exonic CNVs were successfully confirmed (Figure 1), including 48 CNVs (37.5%) with refined exons (Figure S2 and Table S2) and 5 large pathogenic/likely pathogenic CNVs (involving other genes in addition to *DMD*), which were confirmed by chromosomal microarray analysis (CMA) (Figure S3 and Table S3). Overall, the positive predictive value (PPV) of our method for all maternal exonic CNVs was 72.32% (128/177). The PPV for both deletions and duplications was influenced by the size of the exonic CNVs and reached a plateau at sizes of ≥300–1000 kb and ≥1000 kb, respectively (Table 1).

**FIGURE 1:**
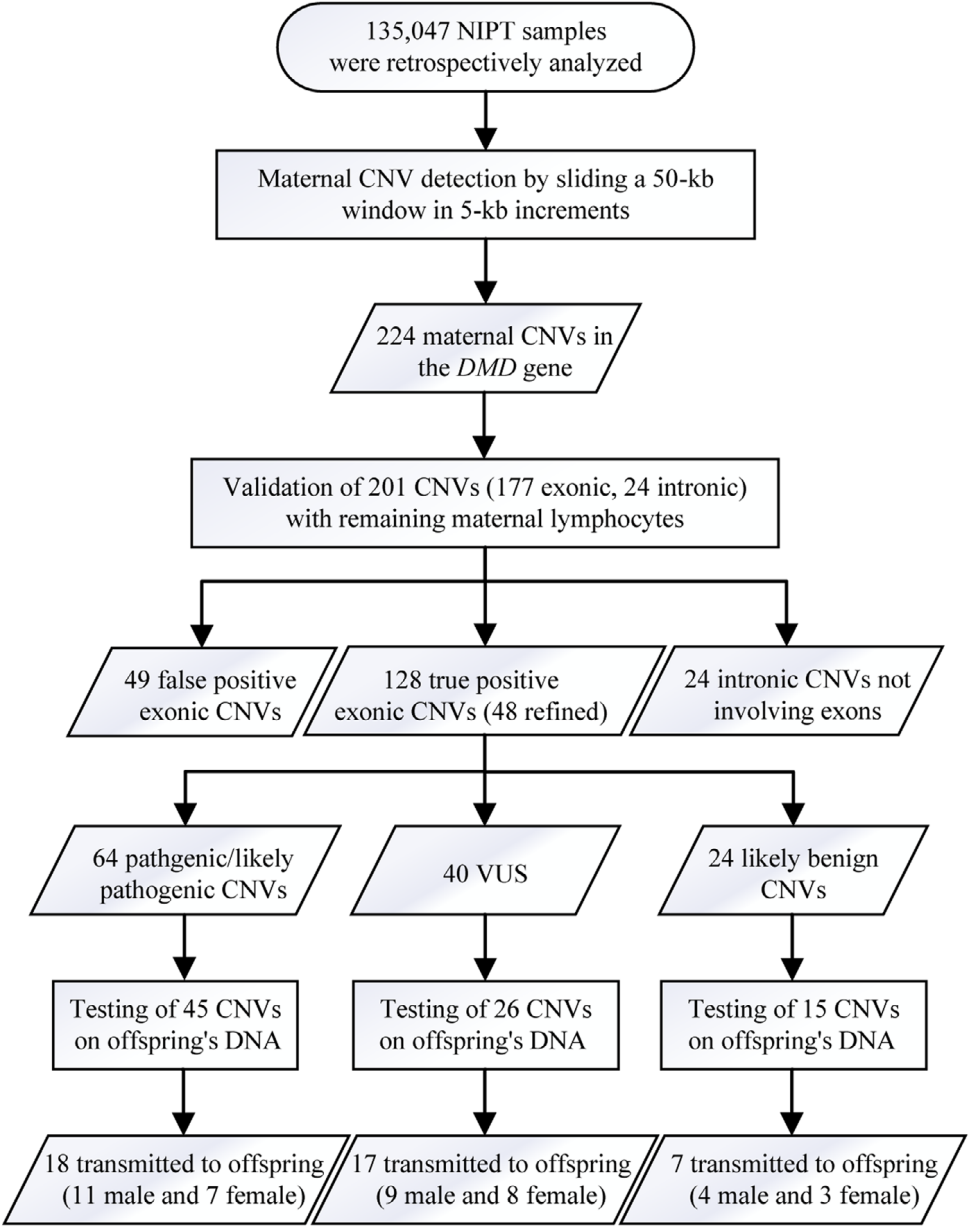
Study design of our study. NIPT, non‐invasive prenatal testing; CNV, copy number variant; VUS, variants of uncertain significance.

**TABLE 1:** Performance of NIPT in maternal CNV analysis for the detection of exonic CNVs in the *DMD* gene.

	NIPT positive	True positive	False positive	No validation	PPV
**Total**	196	128	49	19	72.32%
**Deletion**	109	68	29	12	70.10%
Size within (100–200) kb	64	28	28	8	50.00%
Size within (200–300) kb	18	15	1	2	93.75%
Size within (300‐1000) kb	22	21	0	1	100%
Size ≥1000 kb	5	4	0	1	100%
**Duplication**	87	60	20	7	75.00%
Size within (100–200) kb	14	4	9	1	30.77%
Size within (200–300) kb	9	4	3	2	57.14%
Size within (300–1000) kb	50	39	8	3	82.98%
Size ≥ 1000 kb	14	13	0	1	100%

Abbreviations: CNV, copy number variant; NIPT, non‐invasive prenatal testing; PPV, positive predictive value.

Of these 128 true‐positive maternal exonic CNVs, 64 were classified as pathogenic/likely pathogenic, 40 as variants of uncertain significance (VUS), and 24 as likely benign (Figure 2A and Table 2, Table S4). Among the 64 pathogenic/likely pathogenic CNVs, there were 33 different maternal CNVs in the *DMD* gene, 12 of which were recurrent CNVs. These recurrent CNVs occurred most commonly in exons 48–51, followed by exons 51–52, exons 45–55, and exons 49–51. Using data from the Leiden Open Variation Database,^3^ we further predicted the potential phenotypes for the 64 pathogenic/likely pathogenic maternal CNVs (Figure 2B and Table 2). Overall, 15.63% (10/64) of maternal CNVs detected in the *DMD* gene correlated with the DMD phenotype, 17.19% (11/64) correlated with the likely DMD phenotype, 14.06% (9/64) correlated with the likely BMD phenotype, 42.19% (27/64) correlated with the variable phenotype, and 10.94% (7/64) correlated with the underdetermined phenotype.

**FIGURE 2:**
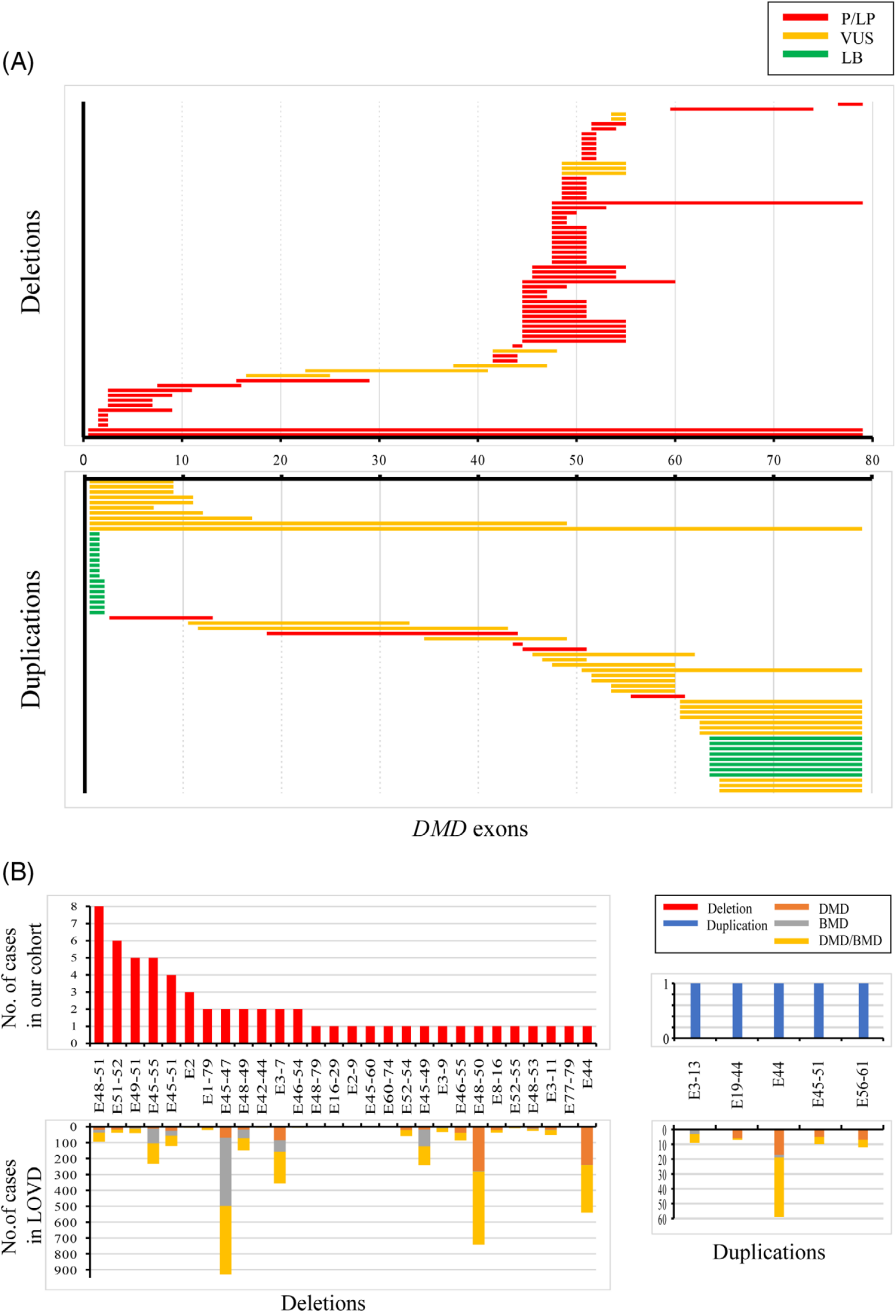
The spectrum of maternal CNVs detected in the *DMD* gene. A) Exon hotspots for the 128 true‐positive maternal exonic CNVs; B) Frequency and phenotypic spectrum of the 64 pathogenic/likely pathogenic CNVs comparing with 19,775 patients from the LOVD. CNVs, copy number variants; P, pathogenic; LP, likely pathogenic; VUS, variants of uncertain significance; LB, likely benign; DMD, Duchenne muscular dystrophy; BMD, Becker muscular dystrophy; LOVD, Leiden Open Variation Database.

**TABLE 2:** Overview of the pathogenic/likely pathogenic true‐positive exonic maternal CNVs identified in 135,047 NIPT samples.

	CNV					Carrier frequency		Inherited status of the offspring	
No.	Del/Dup	Exon	Pathogenicity classification	Phenotype classification^a^	Reading frame	N	1 in _	Tested	Inherited
1	Del	E48‐51	P	Variable	In frame	8	16,881	7	3 (1 M, 2F)
2	Del	E51‐52	P	Likely DMD	In frame	6	22,508	5	2 (2F)
3	Del	E45‐55	P	Likely BMD	In frame	5	27,009	4	1 (1 M)
4	Del	E49‐51	P	Variable	In frame	5	27,009	4	1 (1 M)
5	Del	E45‐51	P	Variable	In frame	4	33,762	4	4 (4 M)
6	Del	E2	P	Underdetermined	Out of frame	3	45,016	2	0
7	Del	E1‐79	P	DMD	Difficult to predict	2	67,524	0	NA
8	Del	E3‐7	P	Variable	Out of frame	2	67,524	1	0
9	Del	E42‐44	P	Variable	In frame	2	67,524	2	0
10	Del	E45‐47	P	Likely BMD	In frame	2	67,524	1	0
11	Del	E46‐54	P	DMD	In frame	2	67,524	2	1 (1 M)
12	Del	E48‐49	P	Variable	In frame	2	67,524	1	0
13	Del	E2‐9	P	Underdetermined	Out of frame	1	135,047	1	1 (1 M)
14	Del	E3‐9	P	Variable	In frame	1	135,047	1	0
15	Del	E3‐11	P	Likely DMD	Out of frame	1	135,047	0	NA
16	Del	E8‐16	P	DMD	Out of frame	1	135,047	1	0
17	Del	E16‐29	P	Variable	In frame	1	135,047	1	0
18	Del	E44	P	Likely DMD	Out of frame	1	135,047	0	NA
19	Del	E45‐49	P	Likely BMD	In frame	1	135,047	1	0
20	Del	E45‐60	P	Likely DMD	In frame	1	135,047	0	NA
21	Del	E46‐55	P	DMD	Out of frame	1	135,047	1	1 (1F)
22	Del	E48‐50	P	Likely DMD	Out of frame	1	135,047	1	1 (1 M)
23	Del	E48‐53	P	Variable	In frame	1	135,047	1	1 (1F)
24	Del	E48‐79	P	Underdetermined	Difficult to predict	1	135,047	0	NA
25	Del	E52‐54	P	DMD	Out of frame	1	135,047	0	NA
26	Del	E52‐55	P	Variable	In frame	1	135,047	1	0
27	Del	E60‐74	LP	Underdetermined	Out of frame	1	135,047	0	NA
28	Del	E77‐79	LP	Underdetermined	Difficult to predict	1	135,047	1	0
29	Dup	E3‐13	P	Likely BMD	In frame	1	135,047	0	NA
30	Dup	E19‐44	P	DMD	In frame	1	135,047	1	1 (1 M)
31	Dup	E44	P	Likely DMD	Out of frame	1	135,047	1	1 (1F)
32	Dup	E45‐51	P	DMD	In frame	1	135,047	0	NA
33	Dup	E56‐61	LP	DMD	Out of frame	1	135,047	0	NA

Abbreviations: BMD, Becker muscular dystrophy; CNV, copy number variant; Del, deletion; DMD, Duchenne muscular dystrophy; Dup, duplication; F, female; LP, likely pathogenic; M, male; N, number of carriers; NA, not available.; NIPT, non‐invasive prenatal testing; P, pathogenic.

^a^ Phenotype classifications were based on the genotype information and patient data of cases from the Leiden Open Variation Database. For detail see materials and methods in supplementary material.

For the 64 women with pathogenic/likely pathogenic variants, further testing was performed for 45 offspring. Overall, 40.00% (18/45) of the offspring inherited true‐positive maternal CNVs from their mothers, including 11 male offspring and 7 female offspring (Figure 1). With a 1:1 male‐to‐female birth ratio, approximately 1.63% (11/67,523.5) of male offspring could be theoretically prenatally diagnosed and managed by our method.

Among the 11 male offspring, 7 were successfully followed up, aged 8 months to 5 years (Table S5). The serum creatine kinase (CK) value in 71.43% (5/7) of these male offspring was elevated (reference 50–310 U/L). In Case M058, we identified a typical out‐of‐frame deletion (exons 48–50), which was classified as pathogenic and transmitted to the male offspring (Figure S4 and Table S5). Although the son manifested as phenotypically normal at 1 year old, a high serum CK level (12,416.8 U/L) suggested the possibility of DMD, which is consistent with our phenotype prediction result (likely DMD). Approximately one‐third of individuals with *DMD* gene variants exhibit neurodevelopmental disorders, with speech delay and learning difficulties being common.^4^ In Case M010, a rare out‐of‐frame deletion (exons 2–9) was detected and transmitted to the male offspring (Figure S4 and Table S5). At two and a half years old, the son showed normal motor development with a normal serum creatine kinase (CK) level (125.4 U/L) but presented delayed speech development (echolalia and slurred speech) and slight behaviour and social problems. To date, this variant has only been reported in a pedigree of monozygotic male twins, in which one manifested autism spectrum disorder and the other presented learning difficulties.^5^ Our study provides further evidence that the deletion of exons 2–9 in the *DMD* gene could be associated with neurodevelopmental disorders rather than DMD/BMD. More relevant studies as well as long‐term follow‐up studies are essential to confirm this finding.

Recent studies in other populations have reported carrier frequencies for *DMD* gene variants, including Belgium (1/2612),^2^ the United States (1/717),^6^ and Israel (1/1046).^7^ Our study extends this knowledge to the Chinese population, revealing an overall carrier frequency of 1/2110 (64/135,047), comparable to that of the Belgian cohort but varying from those of the United States and Israel cohorts. The possible reasons for these differences could be differences in methodologies, the potential influences of race and ethnicity, and different rules of pathogenicity interpretation for variants. Notably, we reported a total of 33 different pathogenic/likely pathogenic maternal CNVs in the *DMD* gene and calculated the carrier frequency for each CNV based on a large general population of reproductive‐age women in China (Table 2), addressing a crucial gap in information regarding the carrier frequency and spectrum of maternal CNVs in the *DMD* gene in the Chinese population. Our datasets provide indispensable information for genetic counselling and can inform policy decisions for DMD/BMD screening worldwide.

In summary, we developed a new strategy for prenatal screening of CNVs in the *DMD* gene based on NIPT data. Using the largest population to date, our study illustrates the spectrum and types of maternal CNVs in the *DMD* gene. Without any additional investment, the new strategy indicated potential clinical significance for maternal CNV detection, which is a natural application expansion for NIPT.

## AUTHOR CONTRIBUTIONS

Lijie Song, Ting Wang, Ping Hu, and Zhengfeng Xu designed the study. Ting Wang, Ping Hu, Xiaojuan Wu, and Ying Xue collected data. Yan Wang, Lulu Meng, Ran Zhou, Jianxin Tan, Dingyuan Ma, Yuguo Wang, Zhu Jiang, and Binbin Shao performed data analysis. Yan Wang, Lulu Meng, Quanze He, and Jingyu Zhao interpreted the data. Yan Wang and Yan Sun generated the bioinformatics workflow. Zhihong Qiao, Zhonghua Wang, Linlin Fan, and Yunmei Chen performed the statistical analyses. Yan Wang, Yan Sun, Lulu Meng, Quanze He, and Jingyu Zhao drafted the manuscript. Lijie Song, Ting Wang, Ping Hu, and Zhengfeng Xu revised the manuscript. All authors read and approved the final manuscript.

## CONFLICT OF INTEREST STATEMENT

The authors declare no conflicts of interest.

## FUNDING INFORMATION

National Key R&D Program of China (No. 2022YFC2703400 to Xu, No. 2022YFC2703400 and No. 2021YFC1005301 to Hu.), the National Natural Science Foundation of China (No. 81971398, 82371862), Jiangsu Province Capability Improvement Project through Science, Technology and Education Jiangsu Provincial Medical Key Discipline (No. ZDXK202211), the Primary Research and Development Plan of Jiangsu Province (BE2022736), and Jiangsu Maternal and Children health care key discipline (FXK202142).

## ETHICS DECLARATION

This study was approved by the Institutional Review Board of Nanjing Maternity and Child Health Care Hospital (2021KY‐069), the Institutional Review Board of BGI (BGI‐IRB 22020) and Suzhou Municipal Hospital (K‐2022‐001‐H01) in accordance with the Helsinki Declaration of 1975, as revised in 2000. Informed consent was obtained from all the study participants at the time of providing samples, and individual data was de‐identified.

## Supporting information

Supporting information

## Data Availability

The datasets supporting the major results/conclusions of this article are included within the article and its supplementary files.
