# Supplementary material for: A new strategy for prenatal genetic screening of copy number variations in the DMD gene: A large cohort study based on NIPT analysis
Source: Clin Transl Med. 2024 May 26;14(5):e1706. doi: 10.1002/ctm2.1706 (PMC11128712; doi:10.1002/ctm2.1706)
Supplement: Supplementary file 1 — Supporting information [file CTM2-14-e1706-s001.docx]

**Supplementary Materials**

**List of Supplementary Information**

**Materials and Methods**

**Supplementary Figures**

**Figure S1.** The age distribution of the population

**Figure S2.** Concordant and discordant position of the 128 true-positive exonic maternal CNVs—NIPT prediction and MLPA validation

**Figure S3.** The strategy for the validation of maternal CNVs in the *DMD* gene identified by NIPT

**Figure S4.** Testing results for 2 representative cases

**Supplementary Tables**

**Table S1.** The list of maternal CNVs detected by maternal CNV analysis in 135,047 NIPT samples

**Table S2.** Size and position of the 128 true-positive exonic maternal CNVs—NIPT prediction and MLPA validation

**Table S3.** Summary of the true-positive exonic maternal CNVs which contained multi genes

**Table S4.** Overview of the VUS/LB true-positive exonic maternal CNVs identified in 135,047 NIPT samples

**Table S5.** Follow up results of these 11 male offspring with pathogenic/likely pathogenic CNVs

**Supplementary** **References**

**Materials and Methods**

**Whole-genome sequencing for NIPT**

Whole-genome sequencing was performed on an Illumina HiSeq2000 platform or a BGISEQ platform for 5-10 million clean single-end reads (35-50 bp). In brief, plasma was purified from whole blood over 8 hours according to a standard dual centrifugation method,^1^ and then cell-free DNA was extracted using a QIAamp Circulating Nucleic Acid Kit (Qiagen Benelux B.V., Venlo, Netherlands) according to the manufacturer’s instructions. Library preparation and sequencing were performed following standard pipelines as previously reported.^1-3^

**Study design and population**

In this study, a self-developed method was implemented for the detection of maternal CNVs using NIPT data from 135,047 pregnant women. Maternal CNVs, especially those clinically relevant to the *DMD* gene, were systematically detected and analyzed in our cohort.

Sequencing data from 135,047 pregnant women who underwent NIPT at Nanjing Maternity and Child Health Care Hospital and Suzhou Municipal Hospital from January 2017 to December 2021 were retrospectively examined. The median age of this cohort was 32 years (range 18-54). Women with a self-reported family history of genetic diseases were excluded. Informed consent for the anonymous analysis of genomic data and possible publication was obtained from all participants.

**Discovery and validation of maternal CNVs in the *DMD* gene**

A local algorithm with minor modifications was used for the detection of maternal CNVs.^4^ Focusing on maternal CNVs larger than 100 kb in the *DMD* gene, all 135,047 NIPT data points were reanalyzed using this method. A local algorithm was used to remove duplicated reads and reads matched at multiple locations. A control set including data on more than 30 whole genomes from plasma samples of normal women was constructed. According to the read depth of our case series, we set 100 kb as the size threshold for maternal CNV detection. CNVs were identified by sliding a 50-kb window in 5-kb increments and calculating the depth ratio between the cases and controls in each sliding window. CNVs with an average copy ratio less than 0.8 were identified as heterozygous deletions, and the ratio needed to be more than 1.2 for duplications.

The remaining maternal lymphocytes from the original peripheral blood samples used for NIPT were used for DNA extraction and the validation of all the detected maternal CNVs in the *DMD* gene. All maternal CNVs involving the *DMD* gene detected by NIPT were first validated using multiplex ligation-dependent probe amplification (MLPA). For true-positive CNVs that contained other genes in addition to *DMD*, pathogenicity was determined according to the 2019 American College of Medical Genetics and Genomics (ACMG)/ClinGen Technical Standards,^5^ and pathogenic/likely pathogenic CNVs were further confirmed by chromosomal microarray analysis (CMA). MLPA was performed using the SALSA MLPA P034 and P035 kits (MRC-Holland, Amsterdam, Netherlands) with maternal lymphocytes as previously described.^6^ CMA was performed using Affymetrix CytoScan 750K array (Affymetrix, Santa Clara, CA) as described.^7^

**Variant classification and phenotype prediction**

After determination of the pathogenicity of CNVs, CNVs were classified into five categories: pathogenic CNVs, likely pathogenic CNVs, variants of uncertain significance (VUS), likely benign CNVs and benign CNVs.^5^

Phenotype prediction in fetuses is challenging. In the current study, phenotype predictions were based on the genotype information and patient data from the Leiden Open Variation Database (LOVD).

LOVD database analysis workflow

In order to further analyze the population frequency of *DMD* variants in our study, we downloaded the “Full data view for gene DMD” database from the LOVD database (https://databases.lovd.nl/shared/variants) and conducted statistical analysis on the samples. The specific steps were as follows:

1. Download the “Full data view for gene DMD” database from the “Variants” section of the LOVD database;

2. Filter the “Exon” label and remove samples with symbol abnormalities, as well as samples that only contain the promoter region or a single intron. Specifically:

1) Remove samples with symbol abnormalities, such as “,”, “-”, “?”;

2) Remove samples that only contain the promoter region, such as _0, 0i, _0_0i, etc.

3) Remove samples that only contain a single intron, such as 14i, 2i, etc.

3. First, extract the CNV type based on the “DNA change (hg19)” label to obtain duplication/deletion information. For samples without information in this label, use the information from the “published as” label. Finally, manually filter samples with conflicting results.

4. Only retain samples with the “Origin” label containing the words “germline” or “de novo”.

5. For the “Disease” label, only include samples with clear DMD, BMD, or DMD/BMD classification.

6. Manually correct samples with doubts and delete samples for which the deletion/duplication status cannot be determined even after manually correction.

7. Obtain the exon region of the variants.

8. Finally, there are 19,775 remaining informative entries, which can be used to calculate the frequency of exon variants in the LOVD database.

Rules for further classify the potential phenotype for the fetuses

For fetuses or newborn infants, it is challenging to classify DMD/BMD due to the absence of phenotypic features. Therefore, we developed a set of phenotype classification rules specifically for fetuses, based on the genotype information and patient data of 19,775 cases from the LOVD database. The specific details of these rules are as follows:

1. DMD: Among the cases with the same exon variant, 100% of them (with more than 5 cases in total) reported DMD phenotype in the LOVD database;

2. BMD: Among the cases with the same exon variant, 100% of them (with more than 5 cases in total) reported BMD phenotype in the LOVD database;

3. Likely DMD: Among the cases with the same exon variant, a) greater than 80% and less than 100% of them reported DMD phenotype in the LOVD database; or b) less than 5 cases reported DMD phenotype in the LOVD database;

4. Likely BMD: Among the cases with the same exon variant, a) greater than 80% and less than 100% of them reported BMD phenotype in the LOVD database; or b) less than 5 cases reported BMD phenotype in the LOVD database;

5. Variable: Among the cases with the same exon variant, greater than 20% and less than 80% of them reported DMD/BMD phenotype in the LOVD database;

6. Underdetermined: No cases with the same exon variant were reported in the LOVD database.

**Clinical follow-up assessment**

For women carrying true-positive maternal CNVs in the DMD gene, further MLPA testing for their offspring was performed if newborn dried blood spot cards were available. Then, the number of affected male offspring was calculated. Clinical follow-up was conducted for male offspring with pathogenic/likely pathogenic CNVs in the DMD gene inherited from their mothers.

The phenotypes of these offspring (if available) were collected by experienced clinicians, including delayed motor and language milestones, muscle wasting and weakness, joint contracture, lumbar lordosis, cardiac impairment, and neurodevelopmental disorders. In addition, these male offspring were encouraged to undergo serum creatine kinase (CK) testing.

**Figure S1.** **The age distribution of the population**


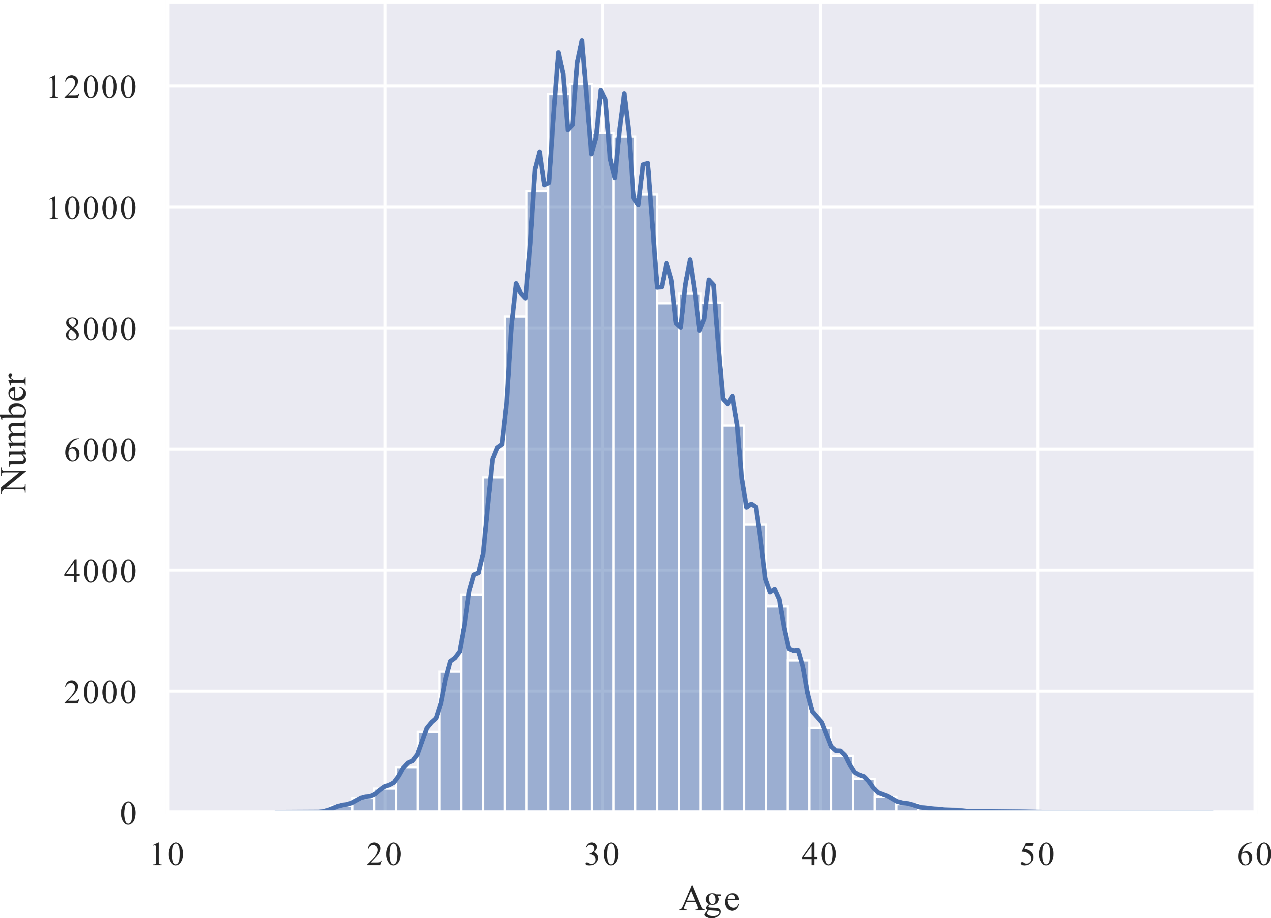


There were 135,047 valid records of samples ranging from 18 to 54 years old, with a mean age of 31 years and a median age of 32 years.

**Figure S2. Concordant and discordant position of the 128 true-positive exonic maternal CNVs—NIPT prediction and MLPA validation**


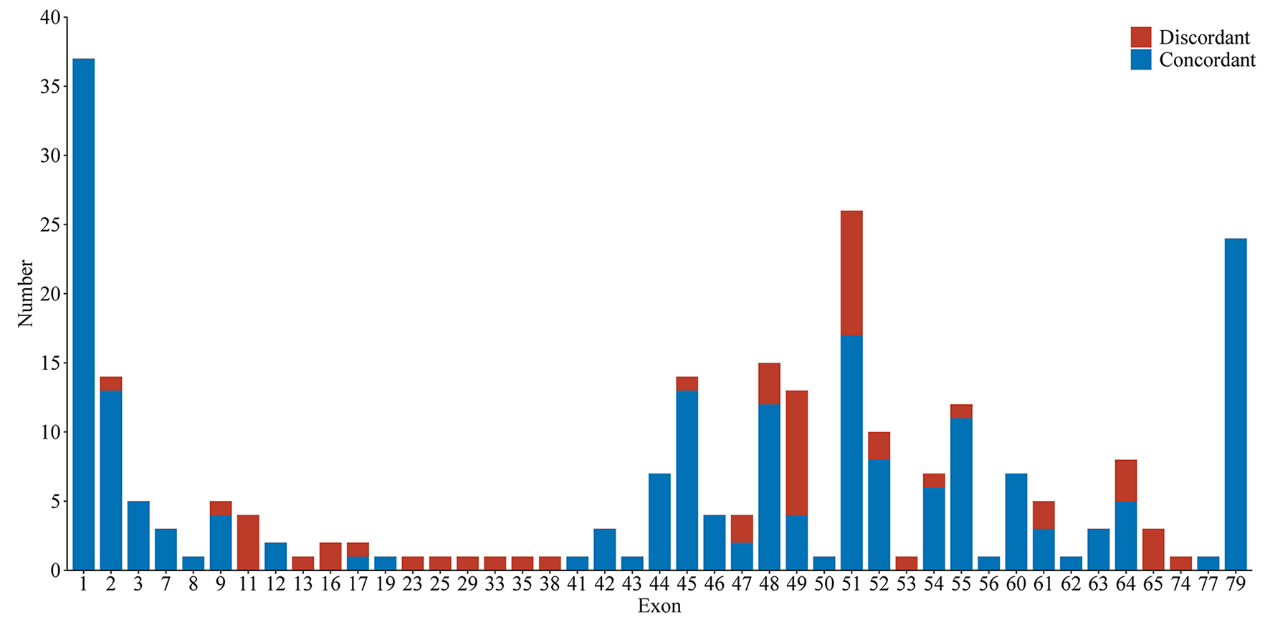


CNVs, copy number variants; NIPT, noninvasive prenatal testing; MLPA, multiplex ligation-dependent probe amplification.

**Figure S3.** **The strategy for the validation of maternal CNVs in the DMD gene identified by NIPT**


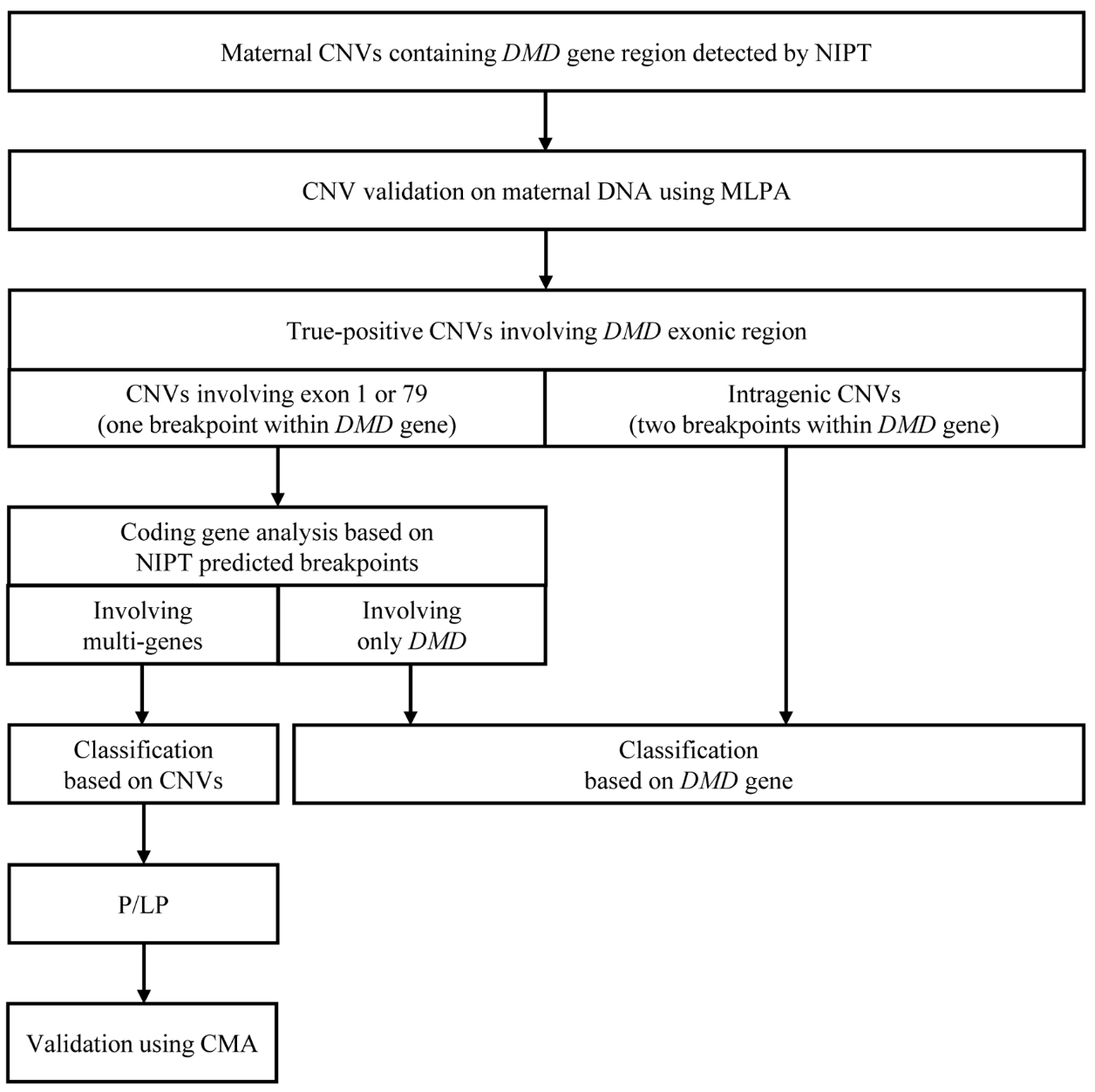


CNV, copy number variant; NIPT, noninvasive prenatal testing; MLPA, multiplex ligation-dependent probe amplification; P, pathogenic; LP, likely pathogenic; CMA, chromosomal microarray analysis.

**Figure S4. Testing results for 2 representative cases**

**
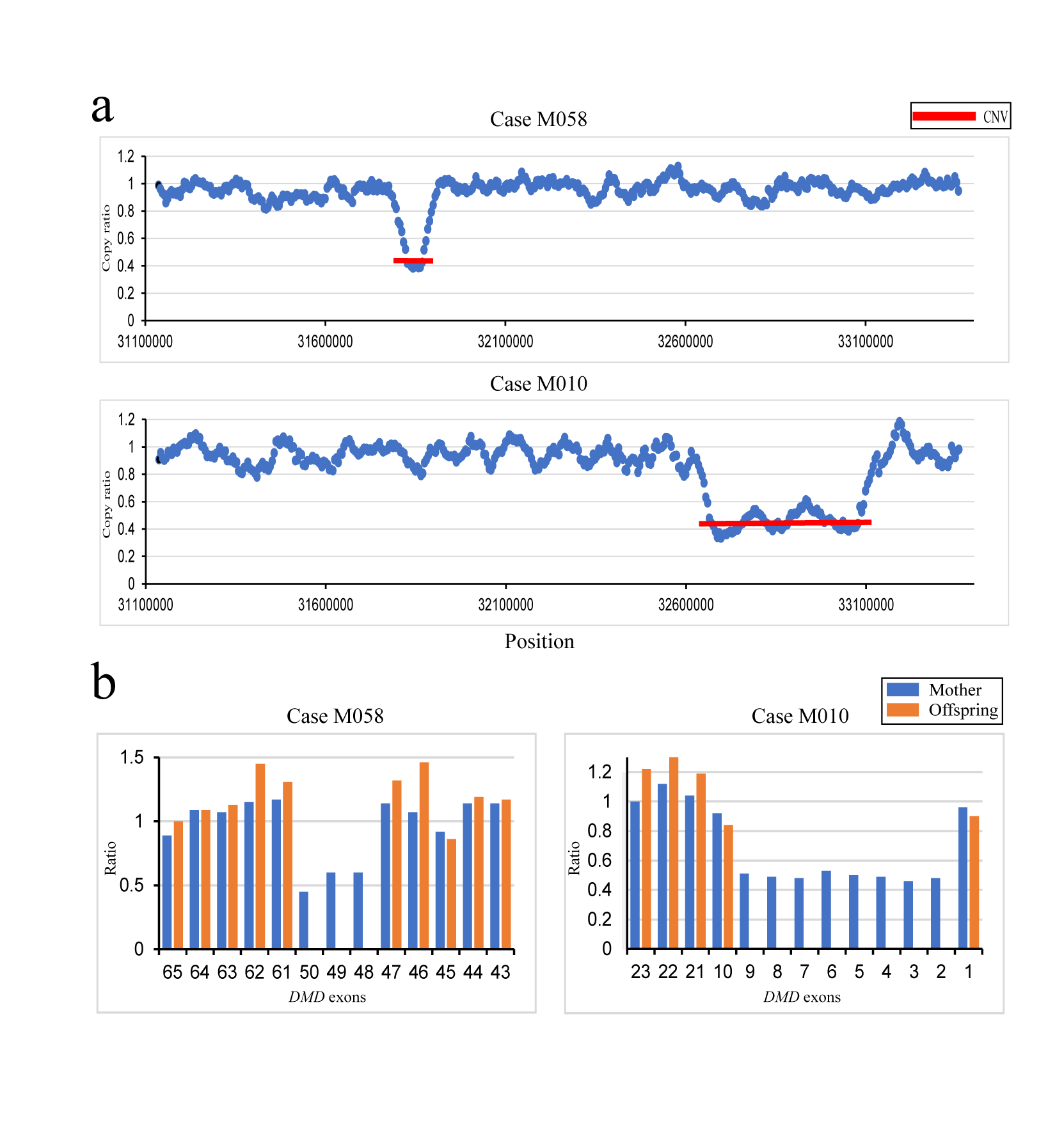
**

a) Noninvasive prenatal testing results of the 2 maternal CNVs for the 2 cases (Case M058 and Case M010); b) MLPA validation for the 2 CNVs. CNVs, copy number variants; MLPA, multiplex ligation-dependent probe amplification.

**Table S1. The list of maternal CNVs detected by maternal CNV analysis in 135,047 NIPT samples**

| **Sample ID** | **Del/Dup** | **NIPT results** | | | | **MLPA results** |
| --- | --- | --- | --- | --- | --- | --- |
|  |  | **Start** | **End** | **Size (kb)** | **Exon/Intron** |  |
| M001 | Del | 5,671,855 | 37,565,302 | 31,893 | E1-79 | E1-79 |
| M002 | Del | 16,386,501 | 36,989,282 | 20,603 | E1-79 | E1-79 |
| M003 | Del | 32,901,869 | 33,093,223 | 191 | E2 | E2 |
| M004 | Del | 32,896,739 | 33,087,906 | 191 | E2 | E2 |
| M005 | Del | 32,943,033 | 33,056,550 | 114 | E2 | Negative |
| M006 | Del | 32,948,261 | 33,056,550 | 108 | E2 | Negative |
| M007 | Del | 32,943,033 | 33,051,304 | 108 | E2 | Negative |
| M008 | Del | 32,917,113 | 33,040,954 | 124 | E2 | Negative |
| M009 | Del | 32,850,308 | 33,130,185 | 280 | E2-4 | E2 |
| M010 | Del | 32,670,809 | 33,151,452 | 481 | E2-9 | E2-9 |
| M011 | Del | 32,770,752 | 32,973,939 | 203 | E3-7 | E3-7 |
| M012 | Del | 32,760,592 | 32,963,714 | 203 | E3-7 | E3-7 |
| M013 | Del | 32,775,828 | 32,891,619 | 116 | E3-7 | Negative |
| M014 | Del | 32,665,761 | 32,953,354 | 288 | E3-9 | E3-9 |
| M015 | Del | 32,629,557 | 32,906,990 | 277 | E3-12 | E3-11 |
| M016 | Del | 32,696,228 | 32,865,781 | 170 | E4-9 | Negative |
| M017 | Del | 32,735,203 | 32,855,500 | 120 | E5-7 | Negative |
| M018 | Del | 32,558,642 | 32,808,949 | 250 | E8-17 | E8-16 |
| M019 | Del | 32,414,297 | 32,614,377 | 200 | E13-30 | E16-29 |
| M020 | Del | 32,476,390 | 32,594,117 | 118 | E14-25 | Negative |
| M021 | Del | 32,461,150 | 32,568,822 | 108 | E17-27 | E17-25 |
| M022 | Del | 32,450,422 | 32,563,689 | 113 | E17-29 | Negative |
| M023 | Del | 32,336,902 | 32,512,255 | 175 | E20-41 | E23-41 |
| M024 | Del | 32,392,685 | 32,502,024 | 109 | E22-34 | Negative |
| M025 | Del | 32,367,442 | 32,476,390 | 109 | E26-37 | Negative |
| M026 | Del | 32,211,615 | 32,392,685 | 181 | E35-44 | Negative |
| M027 | Del | 31,920,806 | 32,382,635 | 462 | E37-47 | E38-47 |
| M028 | Del | 32,007,873 | 32,357,258 | 349 | E42-44 | E42-44 |
| M029 | Del | 32,043,157 | 32,346,976 | 304 | E42-44 | E42-44 |
| M030 | Del | 31,895,025 | 32,346,976 | 452 | E42-47 | E42-48 |
| M031 | Del | 32,048,278 | 32,295,708 | 247 | E44 | E44 |
| M032 | Del | 32,099,416 | 32,237,326 | 138 | E44 | Negative |
| M033 | Del | 31,982,592 | 32,160,643 | 178 | E45 | Negative |
| M034 | Del | 31,972,449 | 32,145,518 | 173 | E45 | Negative |
| M035 | Del | 31,982,592 | 32,124,860 | 142 | E45 | Negative |
| M036 | Del | 31,905,471 | 32,119,725 | 214 | E45-47 | E45-47 |
| M037 | Del | 31,827,723 | 32,150,530 | 323 | E45-50 | E45-49 |
| M038 | Del | 31,822,470 | 32,099,416 | 277 | E45-50 | Negative |
| M039 | Del | 31,770,247 | 32,165,713 | 395 | E45-51 | E45-51 |
| M040 | Del | 31,749,637 | 32,099,416 | 350 | E45-51 | E45-51 |
| M041 | Del | 31,759,904 | 32,073,892 | 314 | E45-51 | E45-51 |
| M042 | Del | 31,754,782 | 32,022,925 | 268 | E45-51 | E45-51 |
| M043 | Del | 31,765,150 | 32,033,068 | 268 | E45-51 | E48-51 |
| M044 | Del | 31,649,951 | 32,058,480 | 409 | E45-54 | E45-55 |
| M045 | Del | 31,604,062 | 32,206,562 | 603 | E45-55 | E45-55 |
| M046 | Del | 31,624,546 | 32,130,022 | 505 | E45-55 | E45-55 |
| M047 | Del | 31,588,785 | 32,038,157 | 449 | E45-55 | E45-55 |
| M048 | Del | 31,560,641 | 31,987,605 | 427 | E45-55 | E45-55 |
| M049 | Del | 31,406,635 | 32,119,725 | 713 | E45-60 | E45-60 |
| M050 | Del | 31,868,738 | 31,982,592 | 114 | E46-48 | E45-47 |
| M051 | Del | 31,879,134 | 31,982,592 | 103 | E46-48 | Negative |
| M052 | Del | 31,727,520 | 31,962,273 | 235 | E46-52 | E48-51 |
| M053 | Del | 31,665,197 | 31,977,469 | 312 | E46-54 | E46-54 |
| M054 | Del | 31,624,546 | 31,982,592 | 358 | E46-55 | E46-55 |
| M055 | Del | 31,634,883 | 31,972,449 | 338 | E46-55 | E46-54 |
| M056 | Del | 31,827,723 | 31,941,031 | 113 | E48-50 | E48-49 |
| M057 | Del | 31,827,723 | 31,935,996 | 108 | E48-50 | E48-49 |
| M058 | Del | 31,812,305 | 31,925,856 | 114 | E48-50 | E48-50 |
| M059 | Del | 31,759,904 | 31,941,031 | 181 | E48-51 | E48-51 |
| M060 | Del | 31,765,150 | 31,925,856 | 161 | E48-51 | E49-51 |
| M061 | Del | 31,780,563 | 31,910,630 | 130 | E48-51 | E48-51 |
| M062 | Del | 31,765,150 | 31,895,025 | 130 | E48-51 | E48-51 |
| M063 | Del | 31,744,564 | 31,930,968 | 186 | E48-52 | E48-51 |
| M064 | Del | 31,727,520 | 31,925,856 | 198 | E48-52 | E48-51 |
| M065 | Del | 31,727,520 | 31,900,420 | 173 | E48-52 | E48-51 |
| M066 | Del | 31,744,564 | 31,900,420 | 156 | E48-52 | E49-51 |
| M067 | Del | 31,665,197 | 31,935,996 | 271 | E48-54 | E48-53 |
| M068 | Del | 31,570,917 | 31,895,025 | 324 | E48-55 | E49-55 |
| M069 | Del | 31,593,801 | 31,895,025 | 301 | E48-55 | E49-55 |
| M070 | Del | 18,485,641 | 31,910,630 | 13,425 | E48-79 | E48-79 |
| M071 | Del | 31,780,563 | 31,889,239 | 109 | E49-51 | E49-51 |
| M072 | Del | 31,749,637 | 31,879,134 | 129 | E49-51 | E49-51 |
| M073 | Del | 31,738,648 | 31,863,654 | 125 | E49-52 | E49-51 |
| M074 | Del | 31,604,062 | 31,884,149 | 280 | E49-55 | E49-55 |
| M075 | Del | 31,712,056 | 31,842,963 | 131 | E50-52 | E51-52 |
| M076 | Del | 31,727,520 | 31,842,963 | 115 | E50-52 | E51-52 |
| M077 | Del | 31,701,786 | 31,817,414 | 116 | E51-52 | E51-52 |
| M078 | Del | 31,706,835 | 31,817,414 | 111 | E51-52 | E51-52 |
| M079 | Del | 31,696,748 | 31,832,866 | 136 | E51-53 | E51-52 |
| M080 | Del | 31,675,519 | 31,837,963 | 162 | E51-54 | E51-52 |
| M081 | Del | 31,649,951 | 31,759,904 | 110 | E52-54 | E52-54 |
| M082 | Del | 31,533,892 | 31,759,904 | 226 | E52-55 | E52-55 |
| M083 | Del | 31,604,062 | 31,738,648 | 135 | E53-55 | Negative |
| M084 | Del | 31,576,227 | 31,732,571 | 156 | E53-55 | Negative |
| M085 | Del | 31,565,690 | 31,696,748 | 131 | E54-55 | E54-55 |
| M086 | Del | 31,565,690 | 31,686,645 | 121 | E54-55 | E54-55 |
| M087 | Del | 31,560,641 | 31,686,645 | 126 | E54-55 | Negative |
| M088 | Del | 31,565,690 | 31,686,645 | 121 | E54-55 | Negative |
| M089 | Del | 31,539,016 | 31,680,546 | 142 | E54-55 | Negative |
| M090 | Del | 31,554,588 | 31,680,546 | 126 | E54-55 | Negative |
| M091 | Del | 31,560,641 | 31,675,519 | 115 | E55 | Negative |
| M092 | Del | 31,488,304 | 31,670,430 | 182 | E55-59 | Negative |
| M093 | Del | 31,462,147 | 31,649,951 | 188 | E55-60 | Negative |
| M094 | Del | 31,131,347 | 31,467,523 | 336 | E60-79 | E60-74 |
| M095 | Del | 31,311,166 | 31,440,295 | 129 | E61-62 | Negative |
| M096 | Del | 31,131,347 | 31,254,632 | 123 | E64-79 | Negative |
| M097 | Del | 26,662,751 | 31,156,911 | 4,494 | E77-79 | E77-79 |
| M098 | Dup | 33,040,954 | 33,985,572 | 945 | E1 | E1 |
| M099 | Dup | 33,051,304 | 33,975,469 | 924 | E1 | E1 |
| M100 | Dup | 33,145,925 | 33,677,124 | 531 | E1 | E1 |
| M101 | Dup | 33,187,832 | 33,671,500 | 484 | E1 | E1 |
| M102 | Dup | 33,119,730 | 33,619,534 | 500 | E1 | E1 |
| M103 | Dup | 33,040,954 | 33,404,239 | 363 | E1 | E1 |
| M104 | Dup | 33,098,651 | 33,388,903 | 290 | E1 | E1 |
| M105 | Dup | 33,172,422 | 33,279,862 | 107 | E1 | E1 |
| M106 | Dup | 33,082,569 | 33,259,393 | 177 | E1 | E1 |
| M107 | Dup | 33,346,905 | 34,143,701 | 797 | E1 | Negative |
| M108 | Dup | 33,295,384 | 33,929,594 | 634 | E1 | Negative |
| M109 | Dup | 33,285,151 | 33,867,727 | 583 | E1 | Negative |
| M110 | Dup | 33,331,676 | 33,739,781 | 408 | E1 | Negative |
| M111 | Dup | 33,311,228 | 33,661,325 | 350 | E1 | Negative |
| M112 | Dup | 33,346,905 | 33,640,979 | 294 | E1 | Negative |
| M113 | Dup | 33,285,151 | 33,614,454 | 329 | E1 | Negative |
| M114 | Dup | 33,285,151 | 33,609,328 | 324 | E1 | Negative |
| M115 | Dup | 33,295,384 | 33,604,238 | 309 | E1 | Negative |
| M116 | Dup | 33,306,118 | 33,541,417 | 235 | E1 | Negative |
| M117 | Dup | 33,306,118 | 33,526,316 | 220 | E1 | Negative |
| M118 | Dup | 33,228,725 | 33,336,804 | 108 | E1 | Negative |
| M119 | Dup | 33,213,357 | 33,326,490 | 113 | E1 | Negative |
| M120 | Dup | 33,020,365 | 34,568,984 | 1,549 | E1-2 | E1-2 |
| M121 | Dup | 32,948,261 | 34,170,072 | 1,222 | E1-2 | E1-2 |
| M122 | Dup | 32,963,714 | 34,036,305 | 1,073 | E1-2 | E1-2 |
| M123 | Dup | 33,004,805 | 34,011,147 | 1,006 | E1-2 | E1-2 |
| M124 | Dup | 32,901,869 | 33,908,837 | 1,007 | E1-2 | E1-2 |
| M125 | Dup | 32,932,745 | 33,677,124 | 744 | E1-2 | E1-2 |
| M126 | Dup | 32,870,850 | 33,614,454 | 744 | E1-2 | E1-2 |
| M127 | Dup | 32,788,212 | 34,446,567 | 1,658 | E1-7 | E1-7 |
| M128 | Dup | 32,717,612 | 34,355,617 | 1,638 | E1-7 | E1-9 |
| M129 | Dup | 32,676,049 | 33,995,819 | 1,320 | E1-9 | E1-9 |
| M130 | Dup | 32,676,049 | 33,990,600 | 1,315 | E1-9 | E1-9 |
| M131 | Dup | 32,624,475 | 33,394,003 | 770 | E1-12 | E1-11 |
| M132 | Dup | 32,624,475 | 33,373,740 | 749 | E1-12 | E1-11 |
| M133 | Dup | 32,614,377 | 33,661,325 | 1,047 | E1-12 | E1-12 |
| M134 | Dup | 32,568,822 | 33,790,865 | 1,222 | E1-16 | E1-17 |
| M135 | Dup | 31,868,738 | 44,207,072 | 12,338 | E1-48 | E1-49 |
| M136 | Dup | 28,231,338 | 41,425,369 | 13,194 | E1-79 | E1-79 |
| M137 | Dup | 32,948,261 | 33,051,304 | 103 | E2 | Negative |
| M138 | Dup | 32,938,006 | 33,046,185 | 108 | E2 | Negative |
| M139 | Dup | 32,865,781 | 32,979,013 | 113 | E3 | Negative |
| M140 | Dup | 32,584,068 | 32,896,739 | 313 | E3-15 | E3-13 |
| M141 | Dup | 32,712,612 | 32,860,553 | 148 | E5-9 | Negative |
| M142 | Dup | 32,717,612 | 32,829,422 | 112 | E7 | Negative |
| M143 | Dup | 32,717,612 | 32,829,422 | 112 | E7 | Negative |
| M144 | Dup | 32,712,612 | 32,824,318 | 112 | E8-9 | Negative |
| M145 | Dup | 32,414,297 | 32,702,399 | 288 | E10-30 | E11-33 |
| M146 | Dup | 32,295,708 | 32,650,592 | 355 | E12-43 | E12-43 |
| M147 | Dup | 32,170,826 | 32,522,644 | 352 | E19-44 | E19-44 |
| M148 | Dup | 31,858,559 | 32,372,532 | 514 | E38-48 | E35-49 |
| M149 | Dup | 32,068,815 | 32,248,650 | 180 | E44 | E44 |
| M150 | Dup | 31,796,703 | 32,140,200 | 343 | E45-50 | E45-51 |
| M151 | Dup | 31,754,782 | 31,952,206 | 197 | E46-51 | E47-51 |
| M152 | Dup | 31,290,491 | 31,972,449 | 682 | E46-62 | E46-62 |
| M153 | Dup | 31,435,115 | 31,920,806 | 486 | E48-60 | E48-60 |
| M154 | Dup | 31,033,930 | 31,796,703 | 763 | E51-79 | E51-79 |
| M155 | Dup | 31,380,493 | 31,775,420 | 395 | E52-60 | E52-60 |
| M156 | Dup | 31,380,493 | 31,770,247 | 390 | E52-60 | E52-60 |
| M157 | Dup | 31,380,493 | 31,691,656 | 311 | E54-60 | E54-60 |
| M158 | Dup | 31,380,493 | 31,686,645 | 306 | E54-60 | E54-60 |
| M159 | Dup | 31,346,986 | 31,604,062 | 257 | E56-61 | E56-61 |
| M160 | Dup | 30,823,702 | 31,549,395 | 726 | E56-79 | E61-79 |
| M161 | Dup | 30,700,584 | 31,445,613 | 745 | E61-79 | E61-79 |
| M162 | Dup | 31,065,211 | 31,445,613 | 380 | E61-79 | E61-79 |
| M163 | Dup | 30,966,963 | 31,351,993 | 385 | E62-79 | E61-79 |
| M164 | Dup | 30,895,594 | 31,321,273 | 426 | E63-79 | E63-79 |
| M165 | Dup | 30,773,564 | 31,295,683 | 522 | E63-79 | E63-79 |
| M166 | Dup | 30,639,291 | 31,285,310 | 646 | E63-79 | E63-79 |
| M167 | Dup | 30,890,505 | 31,326,378 | 436 | E63-79 | E64-79 |
| M168 | Dup | 30,956,736 | 31,285,310 | 329 | E63-79 | E64-79 |
| M169 | Dup | 30,905,691 | 31,280,198 | 375 | E63-79 | E64-79 |
| M170 | Dup | 30,966,963 | 31,264,679 | 298 | E64-79 | E64-79 |
| M171 | Dup | 30,890,505 | 31,264,679 | 374 | E64-79 | E64-79 |
| M172 | Dup | 30,890,505 | 31,264,679 | 374 | E64-79 | E64-79 |
| M173 | Dup | 30,890,505 | 31,259,679 | 369 | E64-79 | E64-79 |
| M174 | Dup | 30,885,415 | 31,259,679 | 374 | E64-79 | E64-79 |
| M175 | Dup | 30,844,337 | 31,269,836 | 425 | E64-79 | E65-79 |
| M176 | Dup | 30,854,517 | 31,218,225 | 364 | E68-79 | E65-79 |
| M177 | Dup | 30,854,517 | 31,203,051 | 349 | E68-79 | E65-79 |
| M178 | Del | 2,699,472 | 58,582,012 | 55,883 | E1-79 | NA |
| M179 | Del | 32,855,500 | 33,135,374 | 280 | E2-4 | NA |
| M180 | Del | 32,702,399 | 33,056,550 | 354 | E2-9 | NA |
| M181 | Del | 32,860,553 | 32,979,013 | 118 | E3-4 | NA |
| M182 | Del | 32,691,171 | 32,808,949 | 118 | E8-9 | NA |
| M183 | Del | 32,336,902 | 32,439,483 | 103 | E30-41 | NA |
| M184 | Del | 32,160,643 | 32,331,836 | 171 | E42-44 | NA |
| M185 | Del | 31,920,806 | 32,027,945 | 107 | E45-47 | NA |
| M186 | Del | 31,744,564 | 31,873,824 | 129 | E49-52 | NA |
| M187 | Del | 31,624,546 | 31,884,149 | 260 | E49-55 | NA |
| M188 | Del | 31,691,656 | 31,801,703 | 110 | E51-53 | NA |
| M189 | Del | 31,521,174 | 31,675,519 | 154 | E55-56 | NA |
| M190 | Dup | 33,130,185 | 33,604,238 | 474 | E1 | NA |
| M191 | Dup | 33,077,364 | 33,249,393 | 172 | E1 | NA |
| M192 | Dup | 2,699,472 | 58,582,012 | 55,883 | E1-79 | NA |
| M193 | Dup | 31,385,811 | 31,749,637 | 364 | E52-60 | NA |
| M194 | Dup | 31,391,109 | 31,598,881 | 208 | E56-60 | NA |
| M195 | Dup | 31,362,732 | 31,619,470 | 257 | E56-61 | NA |
| M196 | Dup | 30,644,573 | 31,244,448 | 600 | E64-79 | NA |
| M197 | Del | 32,917,113 | 33,025,445 | 108 | I2 | Negative |
| M198 | Del | 32,896,739 | 33,015,229 | 118 | I2 | Negative |
| M199 | Del | 32,886,565 | 32,989,359 | 103 | I2 | Negative |
| M200 | Del | 31,987,605 | 32,211,615 | 224 | I44 | Negative |
| M201 | Del | 32,099,416 | 32,206,562 | 107 | I44 | Negative |
| M202 | Del | 32,053,439 | 32,181,018 | 128 | I44 | Negative |
| M203 | Del | 32,048,278 | 32,175,917 | 128 | I44 | Negative |
| M204 | Del | 32,007,873 | 32,170,826 | 163 | I44 | Negative |
| M205 | Del | 32,017,925 | 32,165,713 | 148 | I44 | Negative |
| M206 | Del | 32,002,812 | 32,165,713 | 163 | I44 | Negative |
| M207 | Del | 32,022,925 | 32,160,643 | 138 | I44 | Negative |
| M208 | Del | 32,058,480 | 32,160,643 | 102 | I44 | Negative |
| M209 | Del | 32,017,925 | 32,155,585 | 138 | I44 | Negative |
| M210 | Del | 32,043,157 | 32,155,585 | 112 | I44 | Negative |
| M211 | Del | 32,043,157 | 32,150,530 | 107 | I44 | Negative |
| M212 | Del | 32,027,945 | 32,145,518 | 118 | I44 | Negative |
| M213 | Del | 32,033,068 | 32,145,518 | 112 | I44 | Negative |
| M214 | Del | 32,022,925 | 32,145,518 | 123 | I44 | Negative |
| M215 | Del | 32,022,925 | 32,145,518 | 123 | I44 | Negative |
| M216 | Del | 32,033,068 | 32,135,095 | 102 | I44 | Negative |
| M217 | Del | 31,992,640 | 32,109,421 | 117 | I44 | Negative |
| M218 | Dup | 33,223,637 | 33,331,676 | 108 | I1 | Negative |
| M219 | Dup | 33,046,185 | 33,331,676 | 285 | I1 | Negative |
| M220 | Dup | 33,061,735 | 33,218,402 | 157 | I1 | Negative |
| M221 | Del | 33,061,735 | 33,198,031 | 136 | I1 | NA |
| M222 | Del | 32,033,068 | 32,196,422 | 163 | I44 | NA |
| M223 | Dup | 33,061,735 | 33,182,581 | 121 | I1 | NA |
| M224 | Dup | 31,992,640 | 32,104,416 | 112 | I44 | NA |

CNV, copy number variant; NIPT, noninvasive prenatal testing; Del, deletion; Dup, duplication; MLPA, multiplex ligation-dependent probe amplification, NA, not available.

**Table S2. Size and position of the 128 true-positive exonic maternal CNVs—NIPT prediction and MLPA validation**

| **Category** | **Sample ID** | **Del/Dup** | **NIPT result** | | | | | **MLPA result** | |
| --- | --- | --- | --- | --- | --- | --- | --- | --- | --- |
|  |  |  | **Start** | **End** | **Size (kb)** | **Exon** | **Effect** | **Exon** | **Effect** |
| Completely concordant (N=80) | M001 | Del | 5,671,855 | 37,565,302 | 31,893 | E1-79 | Difficult to predict | E1-79 | Difficult to predict |
|  | M002 | Del | 16,386,501 | 36,989,282 | 20,603 | E1-79 | Difficult to predict | E1-79 | Difficult to predict |
|  | M003 | Del | 32,901,869 | 33,093,223 | 191 | E2 | Out of frame | E2 | Out of frame |
|  | M004 | Del | 32,896,739 | 33,087,906 | 191 | E2 | Out of frame | E2 | Out of frame |
|  | M010 | Del | 32,670,809 | 33,151,452 | 481 | E2-9 | Out of frame | E2-9 | Out of frame |
|  | M011 | Del | 32,770,752 | 32,973,939 | 203 | E3-7 | Out of frame | E3-7 | Out of frame |
|  | M012 | Del | 32,760,592 | 32,963,714 | 203 | E3-7 | Out of frame | E3-7 | Out of frame |
|  | M014 | Del | 32,665,761 | 32,953,354 | 288 | E3-9 | In frame | E3-9 | In frame |
|  | M029 | Del | 32,043,157 | 32,346,976 | 304 | E42-44 | In frame | E42-44 | In frame |
|  | M028 | Del | 32,007,873 | 32,357,258 | 349 | E42-44 | In frame | E42-44 | In frame |
|  | M031 | Del | 32,048,278 | 32,295,708 | 247 | E44 | Out of frame | E44 | Out of frame |
|  | M036 | Del | 31,905,471 | 32,119,725 | 214 | E45-47 | In frame | E45-47 | In frame |
|  | M040 | Del | 31,749,637 | 32,099,416 | 350 | E45-51 | In frame | E45-51 | In frame |
|  | M042 | Del | 31,754,782 | 32,022,925 | 268 | E45-51 | In frame | E45-51 | In frame |
|  | M041 | Del | 31,759,904 | 32,073,892 | 314 | E45-51 | In frame | E45-51 | In frame |
|  | M039 | Del | 31,770,247 | 32,165,713 | 395 | E45-51 | In frame | E45-51 | In frame |
|  | M047 | Del | 31,588,785 | 32,038,157 | 449 | E45-55 | In frame | E45-55 | In frame |
|  | M046 | Del | 31,624,546 | 32,130,022 | 505 | E45-55 | In frame | E45-55 | In frame |
|  | M048 | Del | 31,560,641 | 31,987,605 | 427 | E45-55 | In frame | E45-55 | In frame |
|  | M045 | Del | 31,604,062 | 32,206,562 | 603 | E45-55 | In frame | E45-55 | In frame |
|  | M049 | Del | 31,406,635 | 32,119,725 | 713 | E45-60 | In frame | E45-60 | In frame |
|  | M053 | Del | 31,665,197 | 31,977,469 | 312 | E46-54 | In frame | E46-54 | In frame |
|  | M054 | Del | 31,624,546 | 31,982,592 | 358 | E46-55 | Out of frame | E46-55 | Out of frame |
|  | M058 | Del | 31,812,305 | 31,925,856 | 114 | E48-50 | Out of frame | E48-50 | Out of frame |
|  | M059 | Del | 31,759,904 | 31,941,031 | 181 | E48-51 | In frame | E48-51 | In frame |
|  | M061 | Del | 31,780,563 | 31,910,630 | 130 | E48-51 | In frame | E48-51 | In frame |
|  | M062 | Del | 31,765,150 | 31,895,025 | 130 | E48-51 | In frame | E48-51 | In frame |
|  | M070 | Del | 18,485,641 | 31,910,630 | 13,425 | E48-79 | Difficult to predict | E48-79 | Difficult to predict |
|  | M071 | Del | 31,780,563 | 31,889,239 | 109 | E49-51 | In frame | E49-51 | In frame |
|  | M072 | Del | 31,749,637 | 31,879,134 | 129 | E49-51 | In frame | E49-51 | In frame |
|  | M074 | Del | 31,604,062 | 31,884,149 | 280 | E49-55 | In frame | E49-55 | In frame |
|  | M078 | Del | 31,706,835 | 31,817,414 | 111 | E51-52 | In frame | E51-52 | In frame |
|  | M077 | Del | 31,701,786 | 31,817,414 | 116 | E51-52 | In frame | E51-52 | In frame |
|  | M081 | Del | 31,649,951 | 31,759,904 | 110 | E52-54 | Out of frame | E52-54 | Out of frame |
|  | M082 | Del | 31,533,892 | 31,759,904 | 226 | E52-55 | In frame | E52-55 | In frame |
|  | M086 | Del | 31,565,690 | 31,686,645 | 121 | E54-55 | In frame | E54-55 | In frame |
|  | M085 | Del | 31,565,690 | 31,696,748 | 131 | E54-55 | In frame | E54-55 | In frame |
|  | M097 | Del | 26,662,751 | 31,156,911 | 4,494 | E77-79 | Difficult to predict | E77-79 | Difficult to predict |
|  | M102 | Dup | 33,119,730 | 33,619,534 | 500 | E1 | Difficult to predict | E1 | Difficult to predict |
|  | M100 | Dup | 33,145,925 | 33,677,124 | 531 | E1 | Difficult to predict | E1 | Difficult to predict |
|  | M105 | Dup | 33,172,422 | 33,279,862 | 107 | E1 | Difficult to predict | E1 | Difficult to predict |
|  | M098 | Dup | 33,040,954 | 33,985,572 | 945 | E1 | Difficult to predict | E1 | Difficult to predict |
|  | M099 | Dup | 33,051,304 | 33,975,469 | 924 | E1 | Difficult to predict | E1 | Difficult to predict |
|  | M101 | Dup | 33,187,832 | 33,671,500 | 484 | E1 | Difficult to predict | E1 | Difficult to predict |
|  | M103 | Dup | 33,040,954 | 33,404,239 | 363 | E1 | Difficult to predict | E1 | Difficult to predict |
|  | M104 | Dup | 33,098,651 | 33,388,903 | 290 | E1 | Difficult to predict | E1 | Difficult to predict |
|  | M106 | Dup | 33,082,569 | 33,259,393 | 177 | E1 | Difficult to predict | E1 | Difficult to predict |
|  | M124 | Dup | 32,901,869 | 33,908,837 | 1,007 | E1-2 | Difficult to predict | E1-2 | Difficult to predict |
|  | M121 | Dup | 32,948,261 | 34,170,072 | 1,222 | E1-2 | Difficult to predict | E1-2 | Difficult to predict |
|  | M123 | Dup | 33,004,805 | 34,011,147 | 1,006 | E1-2 | Difficult to predict | E1-2 | Difficult to predict |
|  | M125 | Dup | 32,932,745 | 33,677,124 | 744 | E1-2 | Difficult to predict | E1-2 | Difficult to predict |
|  | M120 | Dup | 33,020,365 | 34,568,984 | 1,549 | E1-2 | Difficult to predict | E1-2 | Difficult to predict |
|  | M122 | Dup | 32,963,714 | 34,036,305 | 1,073 | E1-2 | Difficult to predict | E1-2 | Difficult to predict |
|  | M126 | Dup | 32,870,850 | 33,614,454 | 744 | E1-2 | Difficult to predict | E1-2 | Difficult to predict |
|  | M127 | Dup | 32,788,212 | 34,446,567 | 1,658 | E1-7 | Difficult to predict | E1-7 | Difficult to predict |
|  | M130 | Dup | 32,676,049 | 33,990,600 | 1,315 | E1-9 | Difficult to predict | E1-9 | Difficult to predict |
|  | M129 | Dup | 32,676,049 | 33,995,819 | 1,320 | E1-9 | Difficult to predict | E1-9 | Difficult to predict |
|  | M133 | Dup | 32,614,377 | 33,661,325 | 1,047 | E1-12 | Difficult to predict | E1-12 | Difficult to predict |
|  | M136 | Dup | 28,231,338 | 41,425,369 | 13,194 | E1-79 | Difficult to predict | E1-79 | Difficult to predict |
|  | M146 | Dup | 32,295,708 | 32,650,592 | 355 | E12-43 | In frame | E12-43 | In frame |
|  | M147 | Dup | 32,170,826 | 32,522,644 | 352 | E19-44 | In frame | E19-44 | In frame |
|  | M149 | Dup | 32,068,815 | 32,248,650 | 180 | E44 | Out of frame | E44 | Out of frame |
|  | M152 | Dup | 31,290,491 | 31,972,449 | 682 | E46-62 | In frame | E46-62 | In frame |
|  | M153 | Dup | 31,435,115 | 31,920,806 | 486 | E48-60 | In frame | E48-60 | In frame |
|  | M154 | Dup | 31,033,930 | 31,796,703 | 763 | E51-79 | Difficult to predict | E51-79 | Difficult to predict |
|  | M156 | Dup | 31,380,493 | 31,770,247 | 390 | E52-60 | In frame | E52-60 | In frame |
|  | M155 | Dup | 31,380,493 | 31,775,420 | 395 | E52-60 | In frame | E52-60 | In frame |
|  | M158 | Dup | 31,380,493 | 31,686,645 | 306 | E54-60 | In frame | E54-60 | In frame |
|  | M157 | Dup | 31,380,493 | 31,691,656 | 311 | E54-60 | In frame | E54-60 | In frame |
|  | M159 | Dup | 31,346,986 | 31,604,062 | 257 | E56-61 | Out of frame | E56-61 | Out of frame |
|  | M161 | Dup | 30,700,584 | 31,445,613 | 745 | E61-79 | Difficult to predict | E61-79 | Difficult to predict |
|  | M162 | Dup | 31,065,211 | 31,445,613 | 380 | E61-79 | Difficult to predict | E61-79 | Difficult to predict |
|  | M165 | Dup | 30,773,564 | 31,295,683 | 522 | E63-79 | Difficult to predict | E63-79 | Difficult to predict |
|  | M166 | Dup | 30,639,291 | 31,285,310 | 646 | E63-79 | Difficult to predict | E63-79 | Difficult to predict |
|  | M164 | Dup | 30,895,594 | 31,321,273 | 426 | E63-79 | Difficult to predict | E63-79 | Difficult to predict |
|  | M173 | Dup | 30,890,505 | 31,259,679 | 369 | E64-79 | Difficult to predict | E64-79 | Difficult to predict |
|  | M172 | Dup | 30,890,505 | 31,264,679 | 374 | E64-79 | Difficult to predict | E64-79 | Difficult to predict |
|  | M170 | Dup | 30,966,963 | 31,264,679 | 298 | E64-79 | Difficult to predict | E64-79 | Difficult to predict |
|  | M171 | Dup | 30,890,505 | 31,264,679 | 374 | E64-79 | Difficult to predict | E64-79 | Difficult to predict |
|  | M174 | Dup | 30,885,415 | 31,259,679 | 374 | E64-79 | Difficult to predict | E64-79 | Difficult to predict |
| Incompletely concordant (N=48) | M009 | Del | 32,850,308 | 33,130,185 | 280 | E2-4 | Out of frame | E2 | Out of frame |
|  | M015 | Del | 32,629,557 | 32,906,990 | 277 | E3-12 | In frame | E3-11 | Out of frame |
|  | M018 | Del | 32,558,642 | 32,808,949 | 250 | E8-17 | Out of frame | E8-16 | Out of frame |
|  | M019 | Del | 32,414,297 | 32,614,377 | 200 | E13-30 | In frame | E16-29 | In frame |
|  | M021 | Del | 32,461,150 | 32,568,822 | 108 | E17-27 | In frame | E17-25 | In frame |
|  | M023 | Del | 32,336,902 | 32,512,255 | 175 | E20-41 | Out of frame | E23-41 | In frame |
|  | M027 | Del | 31,920,806 | 32,382,635 | 462 | E37-47 | In frame | E38-47 | In frame |
|  | M030 | Del | 31,895,025 | 32,346,976 | 452 | E42-47 | In frame | E42-48 | In frame |
|  | M037 | Del | 31,827,723 | 32,150,530 | 323 | E45-50 | Out of frame | E45-49 | In frame |
|  | M043 | Del | 31,765,150 | 32,033,068 | 268 | E45-51 | In frame | E48-51 | In frame |
|  | M044 | Del | 31,649,951 | 32,058,480 | 409 | E45-54 | Out of frame | E45-55 | In frame |
|  | M050 | Del | 31,868,738 | 31,982,592 | 114 | E46-48 | Out of frame | E45-47 | In frame |
|  | M052 | Del | 31,727,520 | 31,962,273 | 235 | E46-52 | Out of frame | E48-51 | In frame |
|  | M055 | Del | 31,634,883 | 31,972,449 | 338 | E46-55 | Out of frame | E46-54 | In frame |
|  | M057 | Del | 31,827,723 | 31,935,996 | 108 | E48-50 | Out of frame | E48-49 | in frame |
|  | M056 | Del | 31,827,723 | 31,941,031 | 113 | E48-50 | Out of frame | E48-49 | in frame |
|  | M060 | Del | 31,765,150 | 31,925,856 | 161 | E48-51 | In frame | E49-51 | In frame |
|  | M063 | Del | 31,744,564 | 31,930,968 | 186 | E48-52 | Out of frame | E48-51 | In frame |
|  | M065 | Del | 31,727,520 | 31,900,420 | 173 | E48-52 | Out of frame | E48-51 | In frame |
|  | M064 | Del | 31,727,520 | 31,925,856 | 198 | E48-52 | Out of frame | E48-51 | In frame |
|  | M066 | Del | 31,744,564 | 31,900,420 | 156 | E48-52 | Out of frame | E49-51 | In frame |
|  | M067 | Del | 31,665,197 | 31,935,996 | 271 | E48-54 | Difficult to predict | E48-53 | In frame |
|  | M068 | Del | 31,570,917 | 31,895,025 | 324 | E48-55 | In frame | E49-55 | In frame |
|  | M069 | Del | 31,593,801 | 31,895,025 | 301 | E48-55 | In frame | E49-55 | In frame |
|  | M073 | Del | 31,738,648 | 31,863,654 | 125 | E49-52 | Out of frame | E49-51 | In frame |
|  | M075 | Del | 31,712,056 | 31,842,963 | 131 | E50-52 | Out of frame | E51-52 | In frame |
|  | M076 | Del | 31,727,520 | 31,842,963 | 115 | E50-52 | Out of frame | E51-52 | In frame |
|  | M079 | Del | 31,696,748 | 31,832,866 | 136 | E51-53 | Out of frame | E51-52 | In frame |
|  | M080 | Del | 31,675,519 | 31,837,963 | 162 | E51-54 | Out of frame | E51-52 | In frame |
|  | M094 | Del | 31,131,347 | 31,467,523 | 336 | E60-79 | Difficult to predict | E60-74 | Out of frame |
|  | M128 | Dup | 32,717,612 | 34,355,617 | 1,638 | E1-7 | Difficult to predict | E1-9 | Difficult to predict |
|  | M131 | Dup | 32,624,475 | 33,394,003 | 770 | E1-12 | Difficult to predict | E1-11 | Difficult to predict |
|  | M132 | Dup | 32,619,463 | 33,368,642 | 749 | E1-12 | Difficult to predict | E1-11 | Difficult to predict |
|  | M134 | Dup | 32,568,822 | 33,790,865 | 1,222 | E1-16 | Difficult to predict | E1-17 | Difficult to predict |
|  | M135 | Dup | 31,868,738 | 44,207,072 | 12,338 | E1-48 | Difficult to predict | E1-49 | Difficult to predict |
|  | M140 | Dup | 32,584,068 | 32,896,739 | 313 | E3-15 | In frame | E3-13 | In frame |
|  | M145 | Dup | 32,414,297 | 32,702,399 | 288 | E10-30 | In frame | E11-33 | In frame |
|  | M148 | Dup | 31,858,559 | 32,372,532 | 514 | E38-48 | In frame | E35-49 | In frame |
|  | M150 | Dup | 31,796,703 | 32,140,200 | 343 | E45-50 | Out of frame | E45-51 | In frame |
|  | M151 | Dup | 31,754,782 | 31,952,206 | 197 | E46-51 | Out of frame | E47-51 | In frame |
|  | M160 | Dup | 30,823,702 | 31,549,395 | 726 | E56-79 | Difficult to predict | E61-79 | Difficult to predict |
|  | M163 | Dup | 30,966,963 | 31,351,993 | 385 | E62-79 | Difficult to predict | E61-79 | Difficult to predict |
|  | M168 | Dup | 30,956,736 | 31,285,310 | 329 | E63-79 | Difficult to predict | E64-79 | Difficult to predict |
|  | M169 | Dup | 30,905,691 | 31,280,198 | 375 | E63-79 | Difficult to predict | E64-79 | Difficult to predict |
|  | M167 | Dup | 30,890,505 | 31,326,378 | 436 | E63-79 | Difficult to predict | E64-79 | Difficult to predict |
|  | M175 | Dup | 30,844,337 | 31,269,836 | 425 | E64-79 | Difficult to predict | E65-79 | Difficult to predict |
|  | M177 | Dup | 30,854,517 | 31,203,051 | 349 | E68-79 | Difficult to predict | E65-79 | Difficult to predict |
|  | M176 | Dup | 30,854,517 | 31,218,225 | 364 | E68-79 | Difficult to predict | E65-79 | Difficult to predict |

CNVs, copy number variants; NIPT, noninvasive prenatal testing; Del, deletion; Dup, duplication; MLPA, multiplex ligation-dependent probe amplification, NA, not available.

**Table S3. Summary of the true-positive exonic maternal CNVs which contained multi genes**

| **Sample ID** | **Del/Dup** | **NIPT results** | | | | | **MLPA results** | ***DMD* classification** | **CNV classification** | **CMA results** |
| --- | --- | --- | --- | --- | --- | --- | --- | --- | --- | --- |
|  |  | **Start** | **End** | **Size (kb)** | **Exon** | **Coding Genes** |  |  |  |  |
| M001 | Del | 5,671,855 | 37,565,302 | 31,893 | E1-79 | *DMD* and 128 more | E1-79 | P | P | arr[hg19]Xp22.32p21.1(5,587,367-37,577,972)×1 |
| M002 | Del | 16,386,501 | 36,989,282 | 20,603 | E1-79 | *DMD* and 70 more | E1-79 | P | P | arr[hg19]Xp22.2p21.1(16,388,309-36,989,967)×1 |
| M070 | Del | 18,485,641 | 31,910,630 | 13,425 | E48-79 | *DMD* and 53 more | E48-79 | P | P | arr[hg19]Xp22.13p21.1(18,512,332-31,889,320)×1 |
| M097 | Del | 26,662,751 | 31,156,911 | 4,494 | E77-79 | *DMD* and 15 more | E77-79 | LP | P | arr[hg19]Xp21.3p21.2(26,594,748-31,157,107)×1 |
| M136 | Dup | 28,231,338 | 41,425,369 | 13,194 | E1-79 | *DMD* and 40 more | E1-79 | VUS | P | arr[hg19]Xp21.3p11.4(28,236,259-41,417,165)×3 |
| M127 | Dup | 32,788,212 | 34,446,567 | 1,658 | E1-7 | *DMD, FAM47A* | E1-7 | VUS | VUS | NA |
| M128 | Dup | 32,717,612 | 34,355,617 | 1,638 | E1-7 | *DMD, FAM47A* | E1-9 | VUS | VUS | NA |
| M135 | Dup | 31,868,738 | 44,207,072 | 12,338 | E1-48 | *DMD* and 37 more | E1-49 | VUS | VUS | NA |
| M154 | Dup | 31,033,930 | 31,796,703 | 763 | E51-79 | *DMD, FTHL17* | E51-79 | VUS | VUS | NA |
| M160 | Dup | 30,823,702 | 31,549,395 | 726 | E56-79 | *DMD, FTHL17, TAB3* | E61-79 | VUS | VUS | NA |
| M161 | Dup | 30,700,584 | 31,445,613 | 745 | E61-79 | *DMD, FTHL17, GK, TAB3* | E61-79 | VUS | VUS | NA |
| M162 | Dup | 31,065,211 | 31,445,613 | 380 | E61-79 | *DMD, FTHL17* | E61-79 | VUS | VUS | NA |
| M163 | Dup | 30,966,963 | 31,351,993 | 385 | E62-79 | *DMD, FTHL17, TAB3* | E61-79 | VUS | VUS | NA |
| M164 | Dup | 30,895,594 | 31,321,273 | 426 | E63-79 | *DMD, FTHL17, TAB3* | E63-79 | VUS | VUS | NA |
| M165 | Dup | 30,773,564 | 31,295,683 | 522 | E63-79 | *DMD, FTHL17, TAB3* | E63-79 | VUS | VUS | NA |
| M166 | Dup | 30,639,291 | 31,285,310 | 646 | E63-79 | *DMD, FTHL17, GK, TAB3* | E63-79 | VUS | VUS | NA |
| M175 | Dup | 30,844,337 | 31,269,836 | 425 | E64-79 | *DMD, FTHL17, TAB3* | E65-79 | VUS | VUS | NA |
| M176 | Dup | 30,854,517 | 31,218,225 | 364 | E68-79 | *DMD, FTHL17, TAB3* | E65-79 | VUS | VUS | NA |
| M177 | Dup | 30,854,517 | 31,203,051 | 349 | E68-79 | *DMD, FTHL17, TAB3* | E65-79 | VUS | VUS | NA |
| M120 | Dup | 33,020,365 | 34,568,984 | 1,549 | E1-2 | *DMD, FAM47A* | E1-2 | LB | LB | NA |
| M121 | Dup | 32,948,261 | 34,170,072 | 1,222 | E1-2 | *DMD, FAM47A* | E1-2 | LB | LB | NA |
| M167 | Dup | 30,890,505 | 31,326,378 | 436 | E63-79 | *DMD, FTHL17, TAB3* | E64-79 | LB | LB | NA |
| M168 | Dup | 30,956,736 | 31,285,310 | 329 | E63-79 | *DMD, FTHL17, TAB3* | E64-79 | LB | LB | NA |
| M169 | Dup | 30,905,691 | 31,280,198 | 375 | E63-79 | *DMD, FTHL17, TAB3* | E64-79 | LB | LB | NA |
| M170 | Dup | 30,966,963 | 31,264,679 | 298 | E64-79 | *DMD, FTHL17, TAB3* | E64-79 | LB | LB | NA |
| M171 | Dup | 30,890,505 | 31,264,679 | 374 | E64-79 | *DMD, FTHL17, TAB3* | E64-79 | LB | LB | NA |
| M172 | Dup | 30,890,505 | 31,264,679 | 374 | E64-79 | *DMD, FTHL17, TAB3* | E64-79 | LB | LB | NA |
| M173 | Dup | 30,890,505 | 31,259,679 | 369 | E64-79 | *DMD, FTHL17, TAB3* | E64-79 | LB | LB | NA |
| M1174 | Dup | 30,885,415 | 31,259,679 | 374 | E64-79 | *DMD, FTHL17, TAB3* | E64-79 | LB | LB | NA |

CNV, copy number variant; NIPT, noninvasive prenatal testing; Del, deletion; Dup, duplication; MLPA, multiplex ligation-dependent probe amplification; P, pathogenic; LP, likely pathogenic; VUS, variants of uncertain significance; LB, likely benign; CMA, chromosomal microarray analysis, NA, not available.

**Table S4. Overview of the VUS/LB true-positive exonic maternal CNVs identified in 135,047 NIPT samples**

| **No.** | **CNV** | | **Pathogenicity**  **classification** | **Reading frame** | **Carrier frequency** | | **Inherited status of the offspring** | |
| --- | --- | --- | --- | --- | --- | --- | --- | --- |
|  | **Del/Dup** | **Exon** |  |  | **N** | **1 in _** | **Tested** | **Inherited** |
| 1 | Dup | E1 | LB | Difficult to predict | 9 | 15,005 | 4 | 3 (2M, 1F) |
| 2 | Dup | E64-79 | LB | Difficult to predict | 8 | 16,881 | 6 | 2 (1M, 1F) |
| 3 | Dup | E1-2 | LB | Difficult to predict | 7 | 19,292 | 5 | 2 (1M, 1F) |
| 4 | Dup | E61-79 | VUS | Difficult to predict | 4 | 33,762 | 3 | 1 (1M) |
| 5 | Del | E49-55 | VUS | In frame | 3 | 45,016 | 2 | 2 (1M,1F) |
| 6 | Dup | E1-9 | VUS | Difficult to predict | 3 | 45,016 | 2 | 1 (1M) |
| 7 | Dup | E63-79 | VUS | Difficult to predict | 3 | 45,016 | 3 | 3 (2M, 1F) |
| 8 | Dup | E65-79 | VUS | Difficult to predict | 3 | 45,016 | 3 | 2 (1M,1F) |
| 9 | Del | E54-55 | VUS | In frame | 2 | 67,524 | 2 | 0 |
| 10 | Dup | E1-11 | VUS | Difficult to predict | 2 | 67,524 | 2 | 1 (1F) |
| 11 | Dup | E52-60 | VUS | In frame | 2 | 67,524 | 1 | 1 (1M) |
| 12 | Dup | E54-60 | VUS | In frame | 2 | 67,524 | 2 | 1 (1M) |
| 13 | Del | E17-25 | VUS | In frame | 1 | 135,047 | 0 | NA |
| 14 | Del | E23-41 | VUS | In frame | 1 | 135,047 | 1 | 1 (1M) |
| 15 | Del | E38-47 | VUS | In frame | 1 | 135,047 | 1 | 0 |
| 16 | Del | E42-48 | VUS | In frame | 1 | 135,047 | 1 | 1 (1F) |
| 17 | Dup | E1-7 | VUS | Difficult to predict | 1 | 135,047 | 0 | NA |
| 18 | Dup | E1-12 | VUS | Difficult to predict | 1 | 135,047 | 1 | 1 (1F) |
| 19 | Dup | E1-17 | VUS | Difficult to predict | 1 | 135,047 | 0 | NA |
| 20 | Dup | E1-49 | VUS | Difficult to predict | 1 | 135,047 | 0 | NA |
| 21 | Dup | E1-79 | VUS | Difficult to predict | 1 | 135,047 | 0 | NA |
| 22 | Dup | E11-33 | VUS | In frame | 1 | 135,047 | 0 | NA |
| 23 | Dup | E12-43 | VUS | In frame | 1 | 135,047 | 0 | NA |
| 24 | Dup | E35-49 | VUS | In frame | 1 | 135,047 | 0 | NA |
| 25 | Dup | E46-62 | VUS | In frame | 1 | 135,047 | 1 | 1 (1F) |
| 26 | Dup | E47-51 | VUS | In frame | 1 | 135,047 | 0 | NA |
| 27 | Dup | E48-60 | VUS | In frame | 1 | 135,047 | 1 | 1 (1F) |
| 28 | Dup | E51-79 | VUS | Difficult to predict | 1 | 135,047 | 0 | NA |

VUS, variants of uncertain significance; LB, likely benign; CNV, copy number variant; NIPT, noninvasive prenatal testing; Del, deletion; Dup, duplication; N, number of carriers; M, male; F, female, NA, not available.

**Table S5.** **Follow up results of these 11 male offspring with pathogenic/likely pathogenic CNVs**

| **Sample ID** | **CNV** | | **Pathogenicity classification** | **Phenotype classification^*^** | **Reading frame** | **Family follow-up results** |
| --- | --- | --- | --- | --- | --- | --- |
|  | **Del/Dup** | **Exon** |  |  |  |  |
| M010 | Del | E2-9 | P | Underdetermined | Out of frame | Son carrier age 2.5 years, CK 125.4 U/L, delayed speech development, slight behavior and social problems |
| M039 | Del | E45-51 | P | Variable | In frame | Son carrier age 9 months, CK 586.6 U/L, clinically normal |
| M040 | Del | E45-51 | P | Variable | In frame | Son carrier age 1 year, CK 587 U/L, clinically normal |
| M041 | Del | E45-51 | P | Variable | In frame | NA |
| M042 | Del | E45-51 | P | Variable | In frame | Son carrier age 5 years, CK 844 U/L, clinically normal |
| M047 | Del | E45-55 | P | Likely BMD | In frame | Son carrier age 13 months, CK 2268 U/L, ventricular septal defect identified at 6 months |
| M053 | Del | E46-54 | P | DMD | In frame | NA |
| M058 | Del | E48-50 | P | Likely DMD | Out of frame | Son carrier age 1 year, CK 12416.8 U/L, clinically normal |
| M052 | Del | E48-51 | P | Variable | In frame | NA |
| M066 | Del | E49-51 | P | Variable | In frame | Son carrier age 8 months, CK 272.7 U/L, clinically normal |
| M147 | Dup | E19-44 | P | DMD | In frame | NA |

CNV, copy number variant; Del, deletion; Dup, duplication; P, pathogenic; DMD, Duchenne muscular dystrophy; BMD, Becker muscular dystrophy; CK, creatine kinase, NA, not available.

^*^Phenotype classifications were based on the genotype information and patient data of cases from the Leiden Open Variation Database. For details see materials and methods.

**Supplementary References**

1. Liu H, Gao Y, Hu Z, et al. Performance evaluation of NIPT in detection of chromosomal copy number variants using low-coverage whole-genome sequencing of plasma DNA. *PLoS One*. 2016;11(7):e0159233.

2. Zhang H, Gao Y, Jiang F, et al. Non-invasive prenatal testing for trisomies 21, 18 and 13: clinical experience from 146,958 pregnancies. *Ultrasound Obstet Gynecol*. 2015;45(5):530-8.

3. Ma J, Wang Y, Wang W, et al. Validation of combinatorial probe-anchor ligation-based sequencing as non-invasive prenatal test for trisomy at a central laboratory. *Ultrasound Obstet Gynecol*. 2017;50(1):49-57.

4. Dong Z, Zhang J, Hu P, et al. Low-pass whole-genome sequencing in clinical cytogenetics: a validated approach. *Genet Med*. 2016;18(9):940-8.

5. Riggs ER, Andersen EF, Cherry AM, et al. Technical standards for the interpretation and reporting of constitutional copy-number variants: a joint consensus recommendation of the American College of Medical Genetics and Genomics (ACMG) and the Clinical Genome Resource (ClinGen). *Genet Med*. 2020;22(2):245-257.

6. Zhang J, Ma D, Liu G, et al. Genetic analysis of 62 Chinese families with Duchenne muscular dystrophy and strategies of prenatal diagnosis in a single center. *BMC Med Genet*. 2019;20(1):180.

7. Wang Y, Li Y, Chen Y, et al. Systematic analysis of copy-number variations associated with early pregnancy loss. *Ultrasound Obstet Gynecol*. 2020;55(1):96-104.
